# *ACP4* Variants in Hypoplastic Amelogenesis Imperfecta

**DOI:** 10.1007/s00223-026-01512-y

**Published:** 2026-04-11

**Authors:** Lu Liu, Cheuk Wang Au, Ummey Hany, Alice L. Rigby, Anesha Chauhan, Catriona Brown, Jessie Sims, Gina Murillo, Marìa Gabriela Acosta de Carmargo, Chris F. Inglehearn, Christopher M. Watson, Alan J. Mighell, Claire E. L. Smith

**Affiliations:** 1 School of Dentistry, University of Leeds, Worsley Building, Clarendon Way, Leeds, LS2 9LU UK; 2 Oral Biology, School of Dentistry, University of Leeds, St James’s University Hospital, Leeds, LS9 7TF UK; 3 School of Molecular and Cell Biology, University of Leeds, Worsley Building, Clarendon Way, Leeds, LS2 9LU UK; 4 Birmingham Dental Hospital, Mill Pool Way, Edgbaston, Birmingham, B5 7EG UK; 5 WM RRDN, NIHR Research Delivery Network, Research Park, Vincent Drive, Edgbaston, Birmingham, B15 2TT UK; 6 North East and Yorkshire Genomic Laboratory Hub, Central Lab, St. James’s University Hospital, Leeds, LS9 7TF UK; 7 School of Dentistry, University of Costa Rica, San Pedro, Costa Rica; 8 Department of Paediatric Dentistry, School of Dentistry, Universidad de Carabobo, Valencia, Venezuela; 9 Leeds Institute of Medical Research, University of Leeds, St. James’s University Hospital, Leeds, LS9 7TF UK

**Keywords:** *ACP4*, Amelogenesis imperfecta, Enamel, Acid phosphatase 4

## Abstract

Amelogenesis imperfecta (AI) is a group of rare inherited conditions causing tooth enamel defects. Human acid phosphatase 4 (ACP4) is a transmembrane protein involved in maintaining appositional enamel growth. Variants in *ACP4* cause recessive hypoplastic AI. Here we identify further families and review published *ACP4* variants causing AI. In three Pakistani families, we identified a new *ACP4* variant, c.254T > C, p.(Pro85Leu), which long-read sequencing revealed to be a founder variant. Two further families were homozygous for previously reported pathogenic *ACP4* variants. Further details are also reported for two families previously listed in a technical/cohort study by this group. In total, seventeen *ACP4* variants had been reported in the literature causing AI in seventeen families prior to this study. This report adds an eighteenth variant and brings the total to 22 families. Nine families derive from a cohort of over 400 AI probands curated in Leeds, UK, and account for 9/129 families solved for recessive AI, suggesting *ACP4* variants are a significant cause of recessive AI. *ACP4* variants implicated in AI include fifteen missense, one splice and two frame-breaking deletions. Most missense variants are within the acid phosphatase domain, with one in the transmembrane domain. The consistent hypoplastic phenotype suggests a single mutational mechanism, and the report of a family with a homozygous frameshift variant likely to be subject to nonsense mediated decay points to loss of function. Missense variants alter amino acids at the catalytic core or affect protein stability, homodimerisation or membrane localisation, all likely to result in functional insufficiency.

**Supplementary Information:**

The online version contains supplementary material available at 10.1007/s00223-026-01512-y.

## Background

Human acid phosphatase 4 (ACP4, OMIM*606362) is a member of a family of transmembrane proteins responsible for the hydrolysis of orthophosphoric acid esters [1]. ACP4 serves essential functions only during amelogenesis (enamel formation), although it is also expressed in non-dental tissues [2]. During the secretory stage of amelogenesis, oral epithelium-derived ameloblasts secrete a highly proteinaceous matrix composed of multiple specialised phosphoproteins, including amelogenin, ameloblastin, and enamelin [3]. ACP4 is essential for both initiation and elongation of enamel mineralisation [4]. Previous research has detected acid phosphatase activity to be highest within the Tomes’ process of secretory stage ameloblasts [5], with ACP4 specifically found to localise to the Tomes’ process and lateral membrane of secretory stage ameloblasts [4]. As ameloblasts transition to maturation stage, secretion of enamel matrix proteins (EMPs) ceases and proteolytic enzymes degrade and remove the EMPs, which are transported to lysosomes for further degradation [6]. Concomitantly, hydroxyapatite crystals grow in width and thickness, replacing tissue fluid during maturation [7].

Amelogenesis imperfecta (AI) is a heterogeneous group of rare Mendelian disorders which affect the enamel of both dentitions [8]. Non-syndromic AI represents a disease subgroup in the absence of other morphological or biochemical changes in the body [9]. AI results in abnormalities in enamel structure, appearance and function [10]. Phenotyping AI teeth is challenging due to their wide spectrum of clinical presentation resulting from post-eruptive breakdown [11, 12]. The hypoplastic form is distinguished by quantitative defects in mineralised enamel. Clinical presentations can vary from a pitted appearance to a thinner enamel layer or the complete absence of enamel [12]. By contrast, hypomaturation AI displays normal enamel thickness but with defects in the hardness of enamel with disturbed mineralisation at the maturation stage [13, 14].

Multiple studies have identified autosomal recessive mutations in *ACP4* causing non-syndromic hypoplastic AI (type IJ). To date, 17 families with 17 homozygous or compound heterozygous pathogenic *ACP4* variants have been reported by various groups (Table 1) [4, 15–19]. The majority of variants (14/17) result in missense changes. Kim et al. showed that three out of four pathogenic missense *ACP4* variants that they tested resulted in significantly decreased protein expression and decreased acid phosphatase activity levels, suggesting that *ACP4* variants may cause disease through functional insufficiency [16]. From phenotyping studies of the *Acp4*^R110C/R110C^ mouse model, it has been demonstrated that ACP4 functions during appositional growth of enamel during amelogenesis, although the exact function of ACP4 remains unclear [4].

**Table 1:** Published *ACP4* variants in amelogenesis imperfecta [4, 15–19]

Genomic position	Transcript change	Amino acid change	CADD	gnomAD frequency	Molecular consequence	dbSNP identifier	ACMG	ClinVar	Zygosity	Publication
chr19:50790783	c.226C > T	p.(Arg76Cys)	26.7	0.00001809	Missense	rs1057519277	Path	RCV000415588.3	Hom	Seymen et al
chr19:50790811	c.254 T > C	p.(Leu85Pro)	28.0	0.00003164	Missense	rs762895836	Likely Path	–	Hom	–
chr19:50790819	c.262C > A	p.(Arg88Ser)	24.3	0.000003228	Missense	rs1190557090	Likely Path	RCV001645017.1	C/H	Kim et al
chr19:50791683	c.331C > T	p.(Arg111Cys)	24.2	0.00008120	Missense	rs202073531	Path	RCV000415543.5 RCV002307494.2	Hom /C/H	Seymen et al
chr19:50791702	c.350A > G	p.(Gln117Arg)	23.4	Absent	Missense	rs2123287930	Likely Path	RCV001645018.1	C/H	Kim et al
chr19:50791734	c.382G > C	p.(Ala128Pro)	18.7	0.000006819	Missense	rs767907487	Likely Path	RCV000415614.4	C/H	Seymen et al
chr19:50791749	c.397G > A	p.(Glu133Lys)	21.8	0.00002294	Missense	rs779823931	Likely Path	RCV000415549.3	C/H	Seymen et al
chr19:50791771	c.419C > T	p.(Pro140Leu)	24.5	0.000004344	Missense	rs1371134137	VUS	RCV001645019.1	C/H	Kim et al
chr19:50791780	c.428C > T	p.(Thr143Met)	24.2	0.00004222	Missense	rs546603773	Likely Path	RCV000489568.4	Hom	Smith et al
chr19:50791787	c.433delC	p.(Val146Trpfs*7)	29.0	Absent	Frameshift	rs1295071058	Likely Path	–	C/H	Hany et al
chr19:50792318	c.626 T > C	p.(Leu209Pro)	27.8	Absent	Missense	rs2513790230	VUS	RCV003155004.2	C/H	Bloch-Zupan et al
chr19:50792338	c.645 + 1G > A	?	32.0	0.00000062	Splice	rs1212633515	Path	RCV003154840.2	C/H	Bloch-Zupan et al
chr19:50793751	c.713C > T	p.(Ser238Leu)	26.3	0.000004957	Missense	rs763573828	Likely Path	RCV000415604.3 RCV001643139.1 RCV005355706.1	Hom/ C/H	Seymen et al
chr19:50793774	c.736G > A	p.(Val246Met)	26.2	0.00002107	Missense	rs756274541	Likely Path	RCV003154841.2	C/H	Bloch-Zupan et al
chr19:50793784	c.746C > T	p.(Pro249Leu)	23.8	Absent	Missense	rs1085307111	Likely Path	RCV000489871.4	Hom	Smith et al
chr19:50793812- 50793813	c.774_775del	p.(Gly260Aspfs*29)	33.0	0.00001921	Frameshift	rs768702435	Likely Path	–	Hom	Liang et al
chr19:50793954	c.845 T > C	p.(Met282Thr)	26.7	0.0000006196	Missense	rs2089532683	Likely Path	–	Hom	Hany et al
chr19:50795076	c.1199C > A	p.(Ala400Asp)	23.5	Absent	Missense	rs2513796090	VUS	RCV003154839.2	C/H	Bloch-Zupan et al

Genomic positions are reported according to human reference genome GRCh38/hg38. Variants are reported according to *ACP4* transcript NM_033068.3. ACMG criteria for p.(Leu85Pro) is likely path: likely pathogenic (PP1, pathogenic strong; PM2, pathogenic moderate; PP3, pathogenic supporting). ACMG, American College of Medical Genetics; CADD, combined annotation dependent depletion (v.1.7); C/H, compound heterozygous; ClinVar, public archive of interpretations of clinically relevant variants; dbSNP, single nucleotide polymorphism database (build 157); gnomAD, genome aggregation database (v.4.1) [25]; Het, heterozygous; Hom, homozygous; Path, pathogenic; VUS, variants of uncertain significance

Here we report a novel homozygous missense founder variant and two previously reported variants, causing AI in five families with recessive hypoplastic AI, as well as additional information on two families previously only listed in a technical/cohort study by this group. We also review the mutation spectrum of *ACP4* variants identified in individuals with AI published to date.

## Materials and Methods

### Recruitment and Sample Preparation

Clinical phenotypes were assessed by the recruiting dentist. Participants’ (affected individuals and relatives if available) personal information and blood/saliva for DNA extraction were collected with informed consent obtained, adhering to the principles of the Declaration of Helsinki. Ethical approval was obtained from the Yorkshire and Humber—Leeds East Research Ethics Committee (reference 13/YH/0028; IRAS project ID82448). Genomic DNA was obtained from saliva using Oragene® DNA Sample Collection kits (DNA Genotek, Ottawa, ON, Canada) following the manufacturer’s protocol. One family described herein (Family 4) was screened as part of the UK National Health Service (NHS) R340 (Supplement Table S1) genetic test for AI which uses DNA obtained from blood (https://nhsgms-panelapp.genomicsengland.co.uk/panels/269/v3.0).

### Variant Nomenclature

All variant nomenclature for *ACP4*/ACP4 refers to *ACP4* transcript NM_033068.3 and ACP4 protein sequence NP_149059.1.

### Massively Parallel Sequencing

Proband genomic DNA was processed using the Twist exome comprehensive capture kit (Twist Bioscience, San Francisco, CA, USA), SureSelect All Exons version 5 kit (Agilent, Santa Clara, CA, USA) or using our custom single-molecule molecular inversion probes (smMIPs) protocol as described by Hany et al. [17]. Data were analysed by short-read next generation sequencing of either whole-exome sequencing (WES), or smMIPs data generated on HiSeq 3000, NextSeq 500, or NextSeq 2000 sequencers (Illumina).

WES reads were first processed by Trim Galore (v.0.6.10) and Cutadapt (v.4.4) [20]. The sequences were aligned to the indexed human reference genome hg38 using the BWA software (v.0.7.17) [21]. PCR duplicates were removed using Picard (v.3.1.0) (Broad Institute, 2019). Non-reference bases from 96 samples were combined into cohort data before being genotyped by Genome Analysis Tool Kit (GATK) HaplotypeCaller (v.4.4.0.0) according to recommended best practice workflows [22]. Identified sequence variants were then annotated using Ensembl Variant Effect Predictor (VEP) (v.110.1), along with the population allele frequencies, CADD (v.1.4), and 5’ UTR according to the Genome Aggregation Database (gnomAD; v.3.1.2) and the dbSNP build 138 [23–25]. Annotated data were filtered using VASE (v.0.5.1) (https://github.com/david-a-parry/vase) by chromosomal regions of known and candidate AI genes, CADD score and allele frequency in gnomAD, 1000 Genomes, and dbSNP databases [26, 27]. Variants were filtered for a list of known AI genes and those causative for AI in animal models (Supplement Table S1) reflecting that participants had non-syndromic AI [28]. A minor allele frequency (MAF) of less than 0.01 was used to filter out any variants that were too common in the population to be a plausible cause of recessively inherited AI. Variants with a CADD score (prediction of variant deleteriousness) of less than 15.0 were excluded [24]. Variant pathogenicity status was classified by the updated ClinVar database and the web-based platform Franklin using American College of Medical Genetics and Genomics (ACMG) guidelines [29, 30]. The Linux command line for WES analysis is provided in Supplement.

### Single-Molecule Molecular Inversion Probes (smMIPs) Analysis

The smMIPs data was processed by an in-house bioinformatics pipeline following the manufacturer’s instructions and as previously described by Hany et al. [17].

### Polymerase Chain Reaction (PCR) Amplification of Full Length *ACP4* Fragments

To determine the ancestral haplotype of the c.245 T > C p.(Leu85Pro) *ACP4* variant, long-read sequencing of a 9,647 bp long-range PCR product (spanning chr19:50,788,531–50,798,177) was performed using a GridION sequencer (Oxford Nanopore Technologies (ONT), Oxford, UK).

Each PCR consisted of 1 mL genomic DNA (~ 1–100 ng/mL), 2 mL of 10 × SequalPrep reaction buffer (Invitrogen, Paisley, UK), 0.36 µL of 5 U/µL SequalPrep long polymerase (Invitrogen), 0.4 µL of dimethyl sulfoxide (Invitrogen), 1 µL of 10 × SequalPrep Enhancer A (Invitrogen), 13.24 µL of nuclease free water and 1 µL each of 10 µM forward (dCATCTCCAGGGGCTAGAACG) and reverse (dGGCTCTCAAGGACAGACACC) primers. Thermocycling conditions comprised a denaturation step at 94 °C for 2 min followed by 10 cycles of 94 °C for 10 s, 57 °C for 30 s and 68 °C for 10 min then 20 cycles of 94 °C for 10 s, 57 °C for 30 s and 68 °C for 16 min with an additional 20 s added per cycle, before a final extension step at 72 °C for 5 min. Amplification products were excised from a 0.8% agarose TAE gel and purified using a QIAquick column following manufacturer’s protocols (Qiagen).

To create a nanopore sequencing library using the SQK-LSK114 kit, an end-prep reaction was first performed. This comprised 25 µL of purified amplification product, 3.5 µL Ultra II End-prep Reaction Buffer (New England Biolabs (NEB), Ipswich, Massachusetts, USA) and 1.5 µL of Ultra II End-prep Enzyme Mix (NEB) with the reaction being incubated at 20 °C for 5 min then 65 °C for 5 min. The reaction was cleaned-up with 30 µL of AMPure XP beads (Beckman Coulter, Indianapolis, Indiana, USA) using two 200 µL 80% ethanol washes and eluted in 31 µL of nuclease-free water. Sequencing adapters were ligated to double stranded DNA in a reaction that comprised 30 µL of treated PCR products, 12.5 µL of Ligation Buffer (LNB; ONT), 2.5 µL of Ligation Adapter (LA; ONT) and 5.0 µL of Salt-T4 DNA Ligase (NEB). The reaction was incubated at room temperature for 10 min. An AMPure XP bead (Beckman Coulter) cleanup was next carried out with two 125 µL Long Fragment Buffer washes before the library was eluted using 7 µL of buffer EB (ONT). Flongle flowcells were primed using a solution that comprised 117 µL of Flow Cell Flush (FCF) reagent combined with 3 µL of Flow Cell Tether (FCT) reagent. A sequencing mix comprising 5 µL of the final library, 15 µL of Sequencing Buffer (SB; ONT) and 10 µL of Library Beads (LIB; ONT) was then loaded to the flowcell before a 24-h sequencing run was initiated using MinKNOW software v.24.11.8.

Offline basecalling was performed using Dorado v.1.1.1 (https://github.com/nanoporetech/dorado), with the high accuracy calling model (v.5.2.0), to convert raw pod5 files to FASTQ format. The resulting sequences were selected based on their length (9147–10,147 bp) and quality (Q12) using chopper v.0.11.0 (https://github.com/wdecoster/chopper) [31]. Reads were next aligned to an indexed human reference genome (build GRCh38) using minimap2 v.2.30 (https://github.com/lh3/minimap2), then sorted by alignment coordinate and indexed using samtools v.1.22.1 (http://www.htslib.org/) [32]. Sequencing metrics were evaluated using NanoStat v.1.6.0 (https://github.com/wdecoster/nanostat) and mapped sequenced reads were visualised using the Integrative Genomics Viewer (IGV) v.2.16.2 (https://igv.org/doc/desktop/) [33, 34].

### Sanger Sequencing and Segregation Analyses

Primer pairs were designed using AutoPrimer3 (https://github.com/david-a-parry/autoprimer3) and synthesised by IDT (Leuven, Belgium) to bracket each variant as shown in Supplemental Table S2. Targeted regions were amplified by PCR using Q5® High-Fidelity 2X Master Mix (New England Biolabs, Ipswich, MA, USA). PCR amplicons were evaluated by agarose gel electrophoresis and purified by ExoSAP-IT (ThermoFisher Scientific, Waltham, MA, USA). Sanger sequencing was performed using BigDye Terminator v.3.1 and resolved on the ABI3130xl Genetic Analyser (Applied Biosystems, Paisley, UK). The electropherograms were analysed by 4Peaks (v.1.8) (Nucleobytes).

### Computational Structural Analysis

Computational modelling of ACP4 variants were performed using Rosetta 3.10, as previously described by Au et al. [35]. All calculations were conducted on the University of Leeds AIRE high-performance computing cluster. As there is no crystal structure available for ACP4, an initial model was generated using AlphaFold3 based on the amino acid sequence, and this model served as the starting point for all subsequent modelling [36].

To obtain a low-energy configuration of the structure prior to mutagenesis, the wild-type structure was subjected to the Rosetta Relax protocol. A total of 1000 relaxations were generated, and the lowest-energy model was selected for further modelling. Single point mutations were introduced using the RosettaScripts framework with the *MutateResidue* function. Each mutant structure was subjected to the same scoring function to ensure local backbone and side-chain optimisation. For each variant, 1000 independent models were generated. The scoring function provides Rosetta Energy Unit (REU) scores, and the wildtype REU was subtracted from the mutant REU to obtain the ΔREU for each variant. The locations of the phosphatase active site, signal peptide, and membrane-bound regions were identified using Interpro [37].

## Results

### Dental Phenotyping

**Fig. 1:**
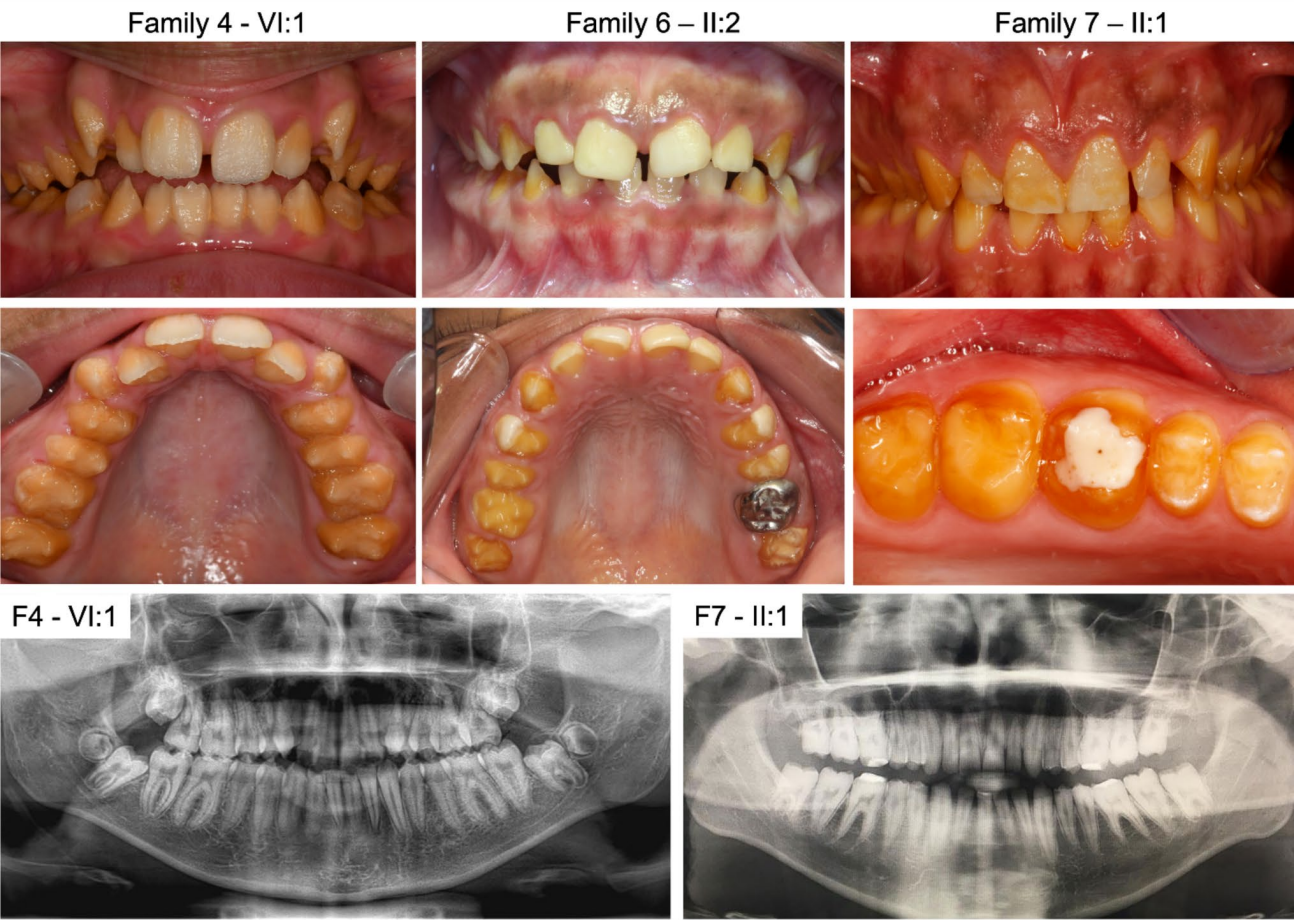
Clinical images and radiographs of teeth from affected individuals. Clinical images from probands F4-V:1, F6-II:2, and F7-II:1 show reduced quantity of enamel on molars with yellow appearance and thin enamel on anterior teeth. Radiographs from probands F4-VI:1 and F7-II:1 illustrate a thin enamel layer on permanent dentition

All probands for which DNA was sequenced from Families 1–7 were diagnosed with hypoplastic AI based on clinical examination and images. Images of teeth from affected individuals from Families 4, 6 and 7 show hypoplastic AI with reduced quantity of enamel in both primary and permanent dentitions. The posterior teeth had yellow discolouration from thin or absent enamel. Anterior teeth were also hypoplastic but to a more limited extent with reduced discolouration and evidence of pitting in some cases (Fig. 1).

### Identification of *ACP4* Variants

**Fig. 2:**
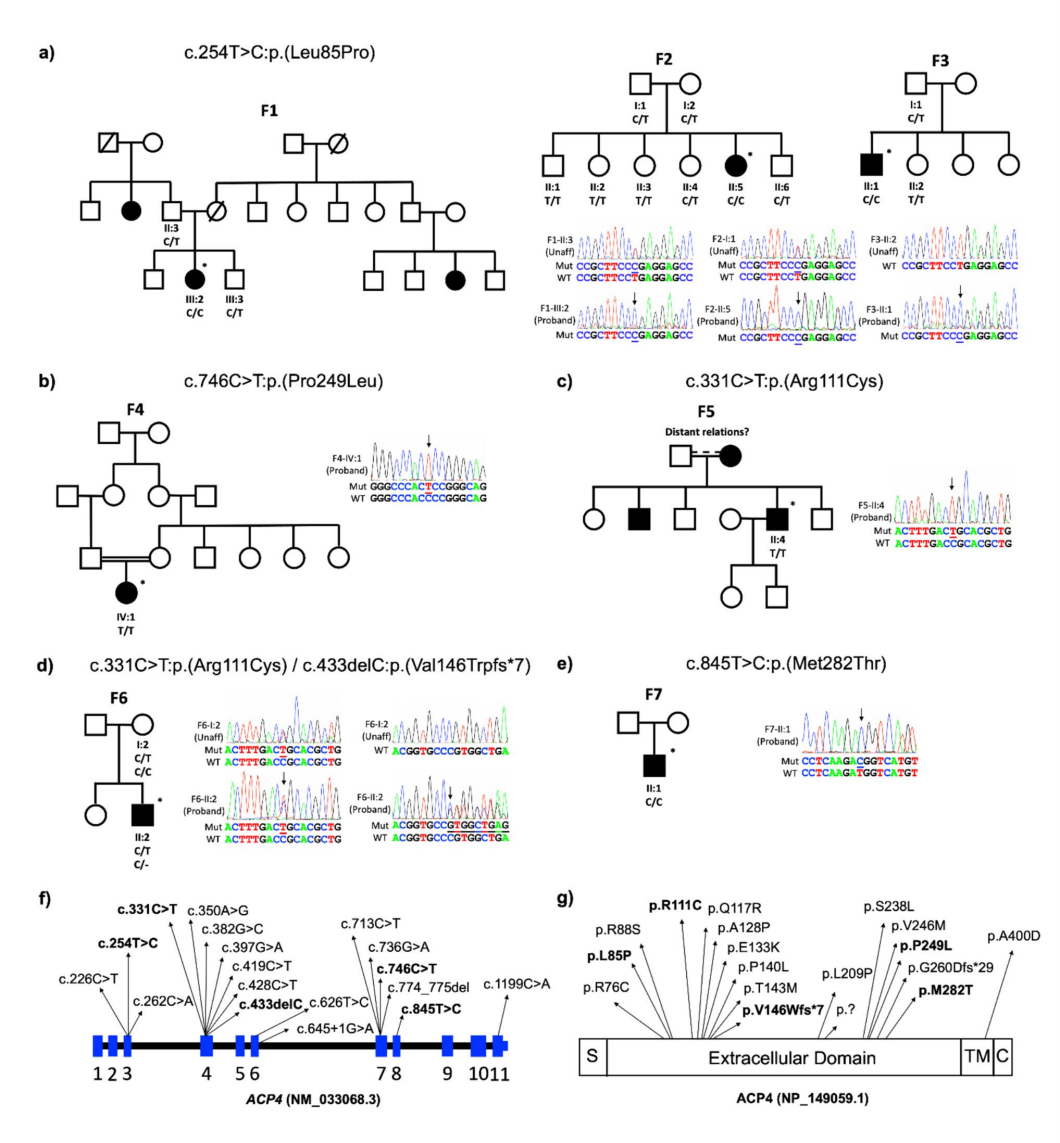
Family pedigrees, variant Sanger sequencing traces and schematic of *ACP4* transcript. Family 1–3 (**a**), c.254 T > C, p.(Leu85Pro) was homozygous in the probands and heterozygous in recruited parental samples. Family 4 (**b**), homozygous c.746C > T, p.(Pro249Leu) identified from consanguineous parents. Family 5 (**c**), homozygous c.331C > T, p.(Arg111Cys) confirmed in proband II:4. Family 6 (**d**), heterozygous c.331C > T, p.(Arg111Cys) confirmed in II:2 and I:2. Heterozygous c.433delC, p.(Val146Trpfs*7) only present in proband II:2. Family 7 (**e**), homozygous c.845 T > C, p.(Met282Thr) was confirmed in proband II:1. (**f**) schematic of all published *ACP4* variants and their locations (NM_033068.3). (**g**) ACP4 domain structure labelled with the position of altered residues from all published variants (NP_149059.2). S = signal peptide; TM = transmembrane domain; C = cytoplasmic domain

Three *ACP4* variants were identified among five families, as a result of on-going screening using various approaches in a cohort of over 400 AI families (Fig. 2). The cohort includes probands with syndromic and non-syndromic AI and is composed both of locally ascertained patients and cases recruited by national and international collaborators.

A novel homozygous variant, c.254T > C, p.(Leu85Pro) in exon 3 of *ACP4*, was identified in three UK families (Families 1–3) of Pakistani origin (Fig. 2a). This alteration is classified as a Variant Of Unknown Significance (VOUS) by Franklin. However, the variant clearly co-segregates with AI in multiple affected family members in each family and given the shared ethnic origin of these families, it is likely they are related, implying further co-segregation with disease between families. When, in light of these findings, criterion PP1 (Pathogenic supporting) is manually changed to strongly supporting, this variant is reclassified likely pathogenic. Furthermore, it is predicted to affect protein function (Sorting Intolerant From Tolerant score = 0.00) [38] and sequence alignments of orthologues indicate that Leu 85 in ACP4 is conserved across 14 mammalian species (Supplement Fig. S1).

The proband in Family 4 was born in the UK from consanguineous parents of Pakistani origin. NHS R340 genetic testing revealed a homozygous *ACP4* variant c.746C > T, p.(Pro249Leu) (rs1085307111), which has been reported previously by Smith et al. in another UK Pakistani consanguineous family (Fig. 2b) [19]. The Family 5 proband was also born from consanguineous parents of UK Pakistani origin. A homozygous pathogenic variant, c.331C > T, p.(Arg111Cys) (rs202073531), previously identified by Seymen et al. in a Turkish family, was identified in proband II:4 following whole exome sequencing analysis (Fig. 2c) [15].

*ACP4* variants identified by smMIPs analysis in Families 6 and 7 were reported in brief as part of a larger cohort analysis by Hany et al. [17], but no family, phenotype or ethnicity details were included. In Family 6, originating from Costa Rica, compound heterozygous *ACP4* variants were identified, including a variant identical to that identified in Family 5, c.331C > T, p.(Arg111Cys) (rs202073531) and a previously unreported frameshift variant, c.433delC, p.(Val146Trpfs*7) (absent in gnomAD) (Fig. 2d). Finally, a previously unreported homozygous missense variant, c.845 T > C, p.(Met282Thr) was identified in the proband of Family 7, who are Venezuelan (Fig. 2e).

A list of all published *ACP4* variants and ACP4 associated AI cases reported to date are reported in Tables 1 and 2 respectively, including those in this report. Their locations on the transcript are shown in Fig. 2f. Positions of the altered amino acid residues are shown in Fig. 2g. All *ACP4* variants identified in this study were submitted to ClinVar, variant accession numbers are SCV000494662, and SCV007338208–SCV007338211.

**Table 2:** Published ACP4-related AI cases/families [4, 15–19]

Family	Origin	*ACP4* Allele 1	*ACP4* Allele 2	References	PMID
1, 2, 3	Turkish	c.713C > T, p.(Ser238Leu)	c.713C > T, p.(Ser238Leu)	Seymen et al. 2016	27843125
4	Turkish	c.331C > T, p.(Arg111Cys)	c.331C > T, p.(Arg111Cys)	Seymen et al. 2016	27843125
5	Turkish	c.226C > T, p.(Arg76Cys)	c.226C > T, p.(Arg76Cys)	Seymen et al. 2016	27843125
6	Turkish	c.382G > C, p.(Ala128Pro)	c.397G > A, p.(Glu133Lys)	Seymen et al. 2016	27843125
7	UK Pakistani	c.428C > T, p.(Thr143Met)	c.428C > T, p.(Thr143Met)	Smith et al. 2017	28513613
8	UK Pakistani	c.746C > T, p.(Pro249Leu)	c.746C > T, p.(Pro249Leu)	Smith et al. 2017	28513613
9	Korean	c.262C > A, p.(Arg88Ser)	c.419C > T, p.(Pro140Leu)	Kim et al. 2022	34036831
10	Turkish	c.350A > G, p.(Gln117Arg)	c.713C > T, p.(Ser238Leu)	Kim et al. 2022	34036831
11	Turkish	c.774_775del, p.(Gly260Aspfs*29)	c.774_775del, p.(Gly260Aspfs*29)	Liang et al. 2022	36183038
12	Turkish	c.713C > T, p.(Ser238Leu)	c.713C > T, p.(Ser238Leu)	Liang et al. 2022	36183038
13	French	c.331C > T, p.(Arg111Cys)	c.645 + 1G > A, p.(?)	Bloch-Zupan et al. 2023	37228816
14	French	c.428C > T, p.(Thr143Met)	c.736 > A, p.(Val246Met)	Bloch-Zupan et al. 2023	37228816
15	French	c.626T > C, p.(Leu209Pro)	c.1199C > A, p.(Ala400Asp)	Bloch-Zupan et al. 2023	37228816
16	Costa Rican	c.331C > T, p.(Arg111Cys)	c.433delC, p.(Val146Trpfs*7)	Hany et al. 2025, this report: Family 6	40741335
17	Venezuelan	c.845T > C, p.(Met282Thr)	c.845T > C, p.(Met282Thr)	Hany et al. 2025, this report: Family 7	40741335
18–20	UK Pakistani	c.254T > C, p.(Leu85Pro)	c.254T > C, p.(Leu85Pro)	This report: Families 1, 2, 3	
21	UK Pakistani	c.746C > T, p.(Pro249Leu)	c.746C > T, p.(Pro249Leu)	This report: Family 4	
22	UK Pakistani	c.331C > T, p.(Arg111Cys)	c.331C > T, p.(Arg111Cys)	This report: Family 5	

The *ACP4* genotypes identified in 22 families with AI. All nomenclature for *ACP4*/ACP4 refers to *ACP4* transcript NM_033068.3 and ACP4 protein sequence NP_149059.1

### Haplotype Analysis Using Long Read Nanopore Sequencing

**Fig. 3:**
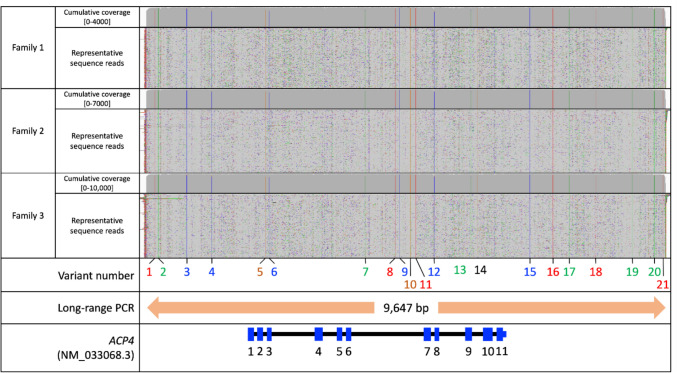
Long-read sequencing analysis of *ACP4.* Integrated Genomics Viewer traces of a 9.6-kb amplification product from the *ACP4* locus spanning exons 1–11 for families 1–3. Nucleotide positions are reported according to human genome build hg38

The presence of the c.254T > C, p.(Leu85Pro) variant in three families from the same geographical area suggests that they may share a common ancestor. This was further investigated by determining the haplotype of flanking variants using long-read PCR. A 9647 bp DNA segment spanning exons 1–11 of *ACP4* was PCR amplified and analysed by long-read sequencing in probands from Families 1, 2, and 3, all of whom were homozygous for c.254T > C, p(Leu85Pro). An identical haplotype consisting of 21 nonreference nucleotides arranged in cis with c.254T > C and spanning at least 9647 bp was shared by the three families analysed, suggesting a shared common ancestor (https://www.ncbi.nlm.nih.gov/bioproject/1403257) (Fig. 3, Table 3).

**Table 3:** Non-reference variants identified as homozygous in Families 1–3 from long-read sequencing of the *ACP4* region

Variant	g.Nomen	c.Nomen	p.Nomen	dbSNP identifier (build 156)	gnomAD frequency (v.4.1) (European Non-Finnish)
1	Chr19(GRCh38):g.50788690C > T	c.-1725C > T		rs56164547	0.41102
2	Chr19(GRCh38):g.50788750G > A	c.-1665G > A		rs59524380	0.41130
3	Chr19(GRCh38):g.50789283T > C	c.-1132T > C		rs57514340	0.23274
4	Chr19(GRCh38):g.50789747G > C	c.-668G > C		rs62114339	0.35479
5	Chr19(GRCh38):g.50790746T > G	c.217-28T > G		rs1978605	0.40409
6	Chr19(GRCh38):g.50790811T > C	c.245T > C	p.(Leu85Pro)	rs762895836	#
7	Chr19(GRCh38):g.50792601C > A	c.645 + 264C > A		rs10425724	0.36049
8	Chr19(GRCh38):g.50793159C > T	c.646-525C > T		rs150423996	0.0053496
9	Chr19(GRCh38):g.50793235T > C	c.646-449T > C		rs266128	0.40714
10	Chr19(GRCh38):g.50793441A > G	c.646-243A > G		rs266127	0.40393
11	Chr19(GRCh38):g.50793539A > T	c.646-145A > T		rs266125	0.39430
12	Chr19(GRCh38):g.50793881T > C	c.779-7T > C		rs45533637	0.019045
13	Chr19(GRCh38):g.50794568_50794569delinsAA	c.973_974delinsAA	p.(Ala325Asn)	rs386810263	Not reported
14	Chr19(GRCh38):g.50794687A > G	c.987-99A > G		rs266123	0.40547
15	Chr19(GRCh38):g.50795655T > C	c.*497T > C		rs266122	0.41071
16	Chr19(GRCh38):g.50796085C > T	c.*927C > T		rs11668998	0.28383
17	Chr19(GRCh38):g.50796388G > A	c.*1230G > A		rs7258708	0.28384
18	Chr19(GRCh38):g.50796882C > T	c.*1724C > T		rs55929248	0.28356
19	Chr19(GRCh38):g.50797562G > A	c.*2404G > A		rs3865442	0.28369
20	Chr19(GRCh38):g.50797973G > A	c.*2815G > A		rs9991	0.26631
21	Chr19(GRCh38):g.50798138C > T	c.*2980C > T	p.( =)	rs4801853	0.28236

Genomic coordinates are according to build GRCh38 of the human reference genome. Transcript numbering is according to the ACP4 MANE transcript NM_033068.3. # The South Asian allele frequency is 0.00058374

Upon analysis of the flanking SNPs identified within *ACP4,* another downstream missense variant, c.973_974delinsAA, p.(Ala325Asn) rs386810263, with a CADD score of 13.98, was identified in all three samples. This variant was not present in gnomAD, but two common missense variants, rs55716643 and rs55735528, were reported causing two different substitutions of the same amino acid, changing it from Ala to Asp or to Thr. These variants have near identical allele frequencies (0.29) and one substitutes the first nucleotide of the triplet, G, for A, while the other substitutes the second nucleotide, a C, for A. Together they would therefore replicate the c.973_974delinsAA variant, suggesting they are in fact one variant, as observed in our sequence data, but have been misclassified as two. If so, this variant has a true frequency of 0.29, excluding it as a cause of disease.

### Structural Modelling

**Fig. 4:**
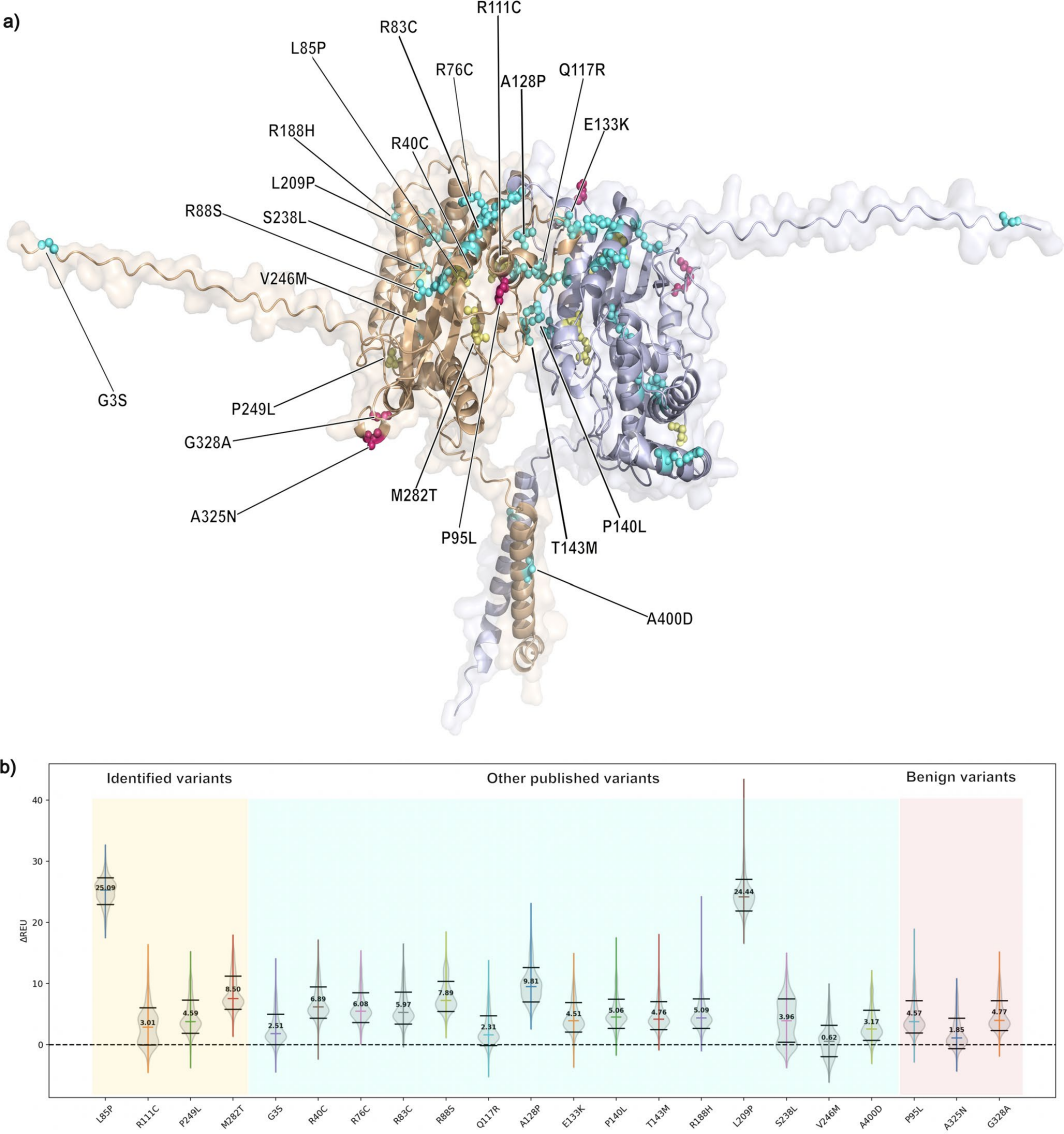
Simulated ACP4 homodimer and mapping of variants. Structural model of the ACP4 homodimer (**a**) highlighting **ACP4** variants identified in this study (yellow), previously reported pathogenic ACP4 variants (blue) and benign ACP4 variants (red). Variants are color-coded to match the violin plot below. **b** Violin graphs of ΔREU are shown for each variant relative to the wild type. Results are shown for all variants identified in this study, all other published ACP4 variants identified in individuals with AI and for three benign variants. Horizontal bars are used to show the standard deviation (black), and the median ΔREU of each variant are displayed. The dashed line at ΔREU = 0 represents the wild type REU

**Fig. 5:**
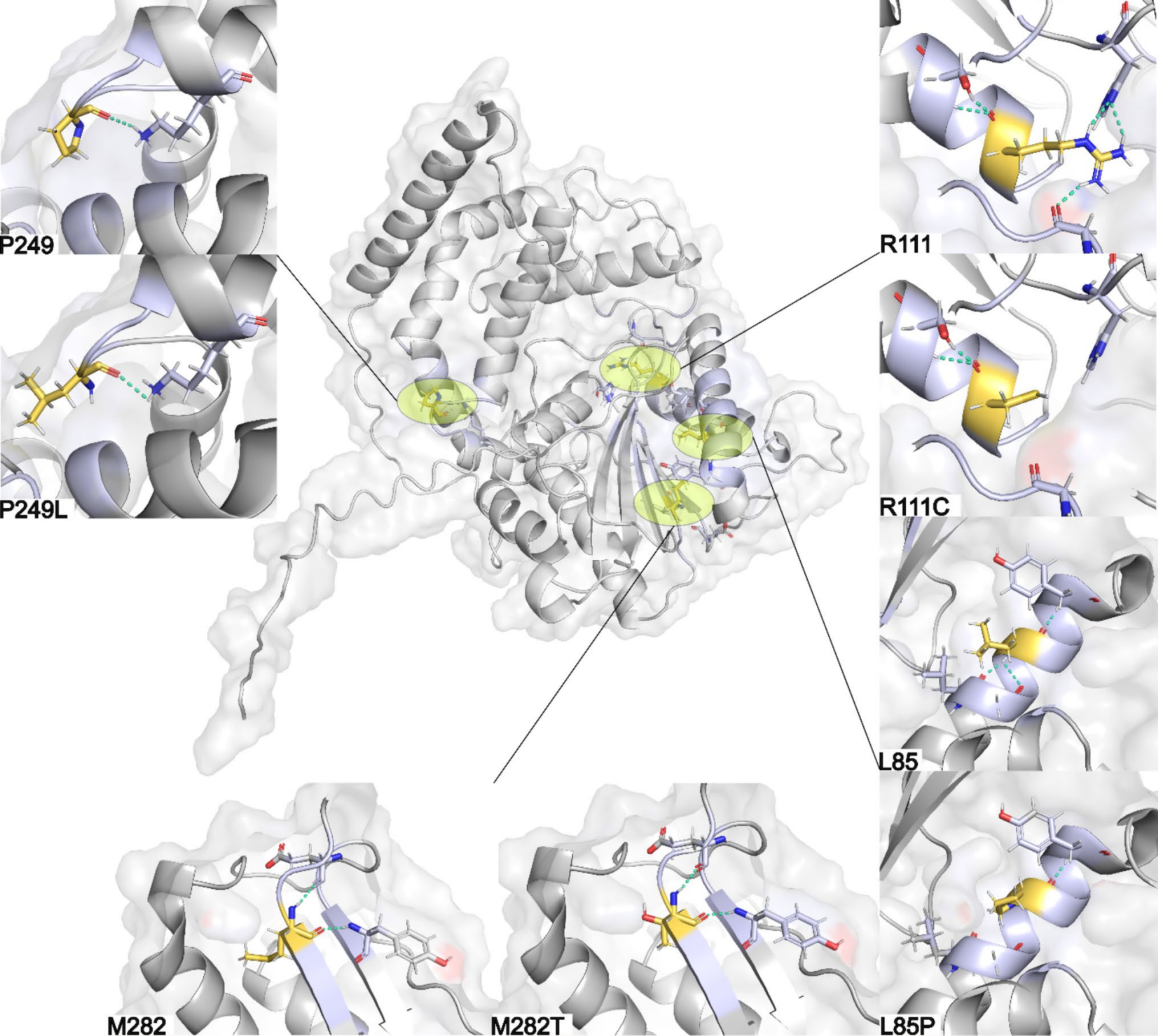
Simulated ACP4 structure and predicted effects of variants. The locations of the four missense variants identified in this study (labelled L85P, R111C, P249L, M282T) are highlighted (yellow) within the AlphaFold3-generated prediction of ACP4 monomer structure. The local environments of wildtype and mutants are displayed using insets (top and bottom, respectively) and side chains are depicted using sticks. Dashed lines indicate hydrogen bonds and polar contacts

Computational modelling was carried out using Rosetta to predict the structural implications of the fifteen likely pathogenic missense *ACP4* variants published here or in previous reports, via their impact on predicted stability. For comparison, we also assessed three *ACP4* missense changes listed in gnomAD but considered too common to be disease causing (allele frequency > 0.05 in gnomAD and the likely misclassified p.(Ala325Asn) variant) and predicted to be benign by Franklin (Supplement Table S3). The Rosetta Energy Units (REU) score resembles kcal/mol, and predicted ΔREU values obtained in silico have been found to be comparable to those obtained experimentally [39]. The substituted amino acids have a range of effects on stability, which are reflected in the ΔREU calculations (Fig. 4). We also assessed the change to the local environment of each substituted amino acid, including predicted interactions with surrounding residues and secondary structures (Fig. 5) and produced a video to show the residue positions substituted as a result of all the pathogenic variants reported to date (10.5518/1814).

Of the variants described in this study, Leu85Pro displayed the strongest destabilisation, due to the disruption of an α-helical region that is important for maintaining fold integrity. Leu85Pro introduces backbone constraints and removes an amide hydrogen required for hydrogen bonding to ensure helix stabilisation [40]. This, in addition to the introduction of a pyrrolidine ring, could lead to a substantial helix distortion. In contrast, Arg111Cys had only slight energetic perturbation, which suggests that the overall structure stability is maintained, despite the loss of a charged side chain. Arg111 is involved in polar contacts but is not buried deeply within the loop, suggesting a structural tolerance for residue substitutions. The change to a cysteine residue likely leads to the loss of some electrostatic interactions.

The effects of Pro249Leu and Met282Thr were observed to be intermediate, or only causing local effects, rather than a total structural collapse. Pro249 enforces loop geometry, and the change to a leucine introduces flexibility, causing loss of backbone rigidity [41]. Pro249Leu also introduces a bulky, hydrophobic side chain into the loop, which could lead to a slight repacking of the protein, inferring a conformational change. Met282Thr introduces a smaller, polar hydroxyl group into a hydrophobic region, which can lead to the exposure of the core to water [42].

Other published variants had varying predicted effects on stability, with the substitutions involving proline, Ala128Pro and Leu209Pro, having the largest impacts due to the introduction of a rigid amino acid with structural constraint.

Modelling of variations predicted to be benign due to their reported allele frequency and/or CADD score was included for comparison with variations predicted to be pathogenic. The median ΔREU value obtained for likely pathogenic variants identified in individuals with AI was 5.06 kcal/mol, whereas for the predicted benign variants, the median value was 4.57 kcal/mol.

## Discussion

Here we describe the identification of a novel homozygous missense founder variant and four additional previously reported variants in *ACP4* (MIM *606,362) in seven families with recessive hypoplastic AI. The homozygous c.254T > C, p.(Leu85Pro) variant, identified in three different UK-Pakistani families (Family 1–3), segregated with AI in all available additional family members. Long-read sequencing confirmed an identical haplotype among three probands from Family 1–3, suggesting they are distantly related. Notably, another mutation, c.713C > T, p.(Ser238Leu), has been detected in four Turkish families to date, suggesting that this *ACP4* variant may also be a founder variant within that population [15, 16]. The other four variants identified include previously published variants, c.746C > T, p.(Pro249Leu) (rs1085307111) identified as biallelic in Family 4 and c.331C > T, p.(Arg111Cys) (rs202073531) identified in as biallelic in Family 5 and as heterozygous in Family 6 [15, 19]. The p.(Pro249Leu) variant has now been identified in two British families of Pakistani heritage to date and the p.(Arg111Cys) variant has been identified in 3 families, including those of Turkish, French and Costa Rican heritage. These findings add to the current mutation spectrum for ACP4 and further underline its critical role in pathological amelogenesis, although the function of this phosphatase during enamel formation remains unclear [16].

To date, including families reported here, 22 families with AI due to segregating *ACP4* pathogenic variants have been reported in the literature, summarised in Table 2. Among the nine families with *ACP4* variants identified in Leeds, seven are of Pakistani origin, one Costa Rican and one Venezuelan. This reflects a bias in the Leeds AI cohort towards local families of Pakistani heritage, which results primarily from the increased incidence of consanguinity and the resulting recessive disease in the Bradford Asian community [43]. Previously published reports of *ACP4* AI reflect a wide heritage. Accordingly, *ACP4* variants can be expected to be encountered as a cause of recessive AI. Current understanding is that *ACP4* variants are not causative of disease in other tissues and organs, unlike many other genes causative for recessive hypoplastic AI. As additional individuals with AI due to *ACP4* variants are identified, including via state-driven genomic testing, it will become clearer if there is ever an impact beyond enamel formation. Improved understanding of the optimum patient pathway for affected individuals will follow.

All variants appear, from the reported hypoplastic enamel phenotypes, to perturb the secretory phase of amelogenesis. However, the individual who carried compound heterozygous *ACP4* variants: c.626T > C, p.(Leu209Pro);c.1199C > A, p.(Ala400Asp), also had agenesis of teeth 18 and 28, consistent with a *WNT10A* phenotype. The individual also carries a heterozygous *WNT10A* variant: NM_025216.3:c.682T > A, p.(Phe228Ile). In addition to selective tooth agenesis, *WNT10A* variants have also been tentatively associated with hypoplastic enamel phenotypes in mice, although the effect might result from reduced dentine volume [44]. Of the *ACP4* variants reported to date, c.626T > C, p.(Leu209Pro), c.1199C > A, p.(Ala400Asp) and the intron 6 splice variant c.645 + 1G > A have only been reported as part of a large cohort study which did not detail family pedigrees, nor confirm segregation [18]. Therefore, the causal association of the *ACP4* c.626T > C, p.(Leu209Pro) and c.1199C > A, p.(Ala400Asp) variants with AI require further validation.

In all, fifteen missense, one splice and two frame-breaking deletion *ACP4* pathogenic variants have been identified. Most variants are clustered in exons 3 (3 missense variants), 4 (6 missense, 1 frameshift) and 7 (3 missense, 1 frameshift) in the histidine phosphatase domain, with all but one variant (c.1199C > A, p.(Ala400Asp)) located within the extracellular portion of the protein. The ACP4 protein includes a signal peptide (amino acids 1–28), a large extracellular domain, making up the majority of the protein (amino acids 29–390), a transmembrane domain (amino acids 391–414) and a cytoplasmic domain (amino acids 415–426) [15]. ACP4 also contains some highly conserved residues likely critical to its structure and function that include those that make up the sites of disulphide bonds: Cys159:Cys378, Cys214:Cys312, Cys353:Cys357, the N-glycosylation sites: Asn191, Asn269, Asn330, Asn339 and the regions surrounding the catalytic residues, denoted as the histidine acid phosphatase active sites: amino acids 32–46 and 282–298 [15].

It has been suggested that biallelic *ACP4* variants causing AI do so through functional insufficiency [16]. One possible mechanism by which amino-acid substitutions can cause loss of function is through alterations in protein stability and/or binding interactions. To test for alterations in protein stability, we performed computational modelling of the fifteen likely pathogenic missense *ACP4* variants and, for comparison, three likely ACP4 missense polymorphisms, using Rosetta 3.10 [35]. Rosetta’s *MutateResidue* protocol and associated scoring function, which evaluate the impact of amino acid substitutions on protein stability, were used in this study. This analysis suggests that protein stability does play a role in AI disease causation for some, but not all, pathogenic variants, with a range of effects varying from no effect on stability to very substantial changes. The median score for disease causing variants was 5.06 kcal/mol, while benign variants were predicted to have less impact on stability, with a median score of 4.57 kcal/mol. This indicates that these residues may be more tolerant to substitutions, but the relatively similar value may suggest that protein stability is only significantly affected by particular variants which are also more likely to exert a pathogenic effect. Consequently, it should be noted that alterations in binding affinity are not captured in the values obtained from this analysis. Binding interactions are also likely to be important to its proper function. ACP4 is also known to function as a homodimer, with residues at the interface between the monomers important to its structure and residues at its catalytic core fundamental to its function [15, 16].

The extreme destabilisation predicted for c.254T > C, p.(Leu85Pro) and c.626T > C, p.(Leu209Pro) is likely to cause loss of function due to the inclusion of a proline residue, which would be expected to disrupt wild-type secondary structure by affecting folding or stability of the protein. Conversely, Arg111Cys is predicted to maintain stability similar to wild-type. However, the InterPro Protein family database predicts that residues 41, 44, 111, 288 and 289 make up the catalytic core, meaning that even minor changes in local electrostatic interactions or changes of binding interactions at residue 111 are likely to be sufficient to cause loss of function (European Bioinformatics Institute (EMBL-EBI), http://www.ebi.ac.uk/) [45]. The c.1199C > A, p.(Ala400Asp) also shows only a minor effect on stability, but this variant, which is in the transmembrane domain (residues 391–414), replaces a hydrophobic alanine with a hydrophilic aspartic acid, and may therefore disrupt insertion of the protein into the membrane. Overall, these findings are therefore consistent with a functional insufficiency mechanism and suggest that the missense variants documented have a range of different effects on the ACP4 protein.

Phenotypically, ACP4-related AI teeth revealed a generalised hypoplastic appearance. Clinical information suggests a characteristic yellow appearance of the posterior molars, with evidence of striated or pitted enamel present on the incisors. To categorise phenotypes of ACP4-related AI accurately, radiographs before eruption are required to measure the enamel volume and exclude any post-eruptive breakdown of the enamel. A previous study examining an extracted tooth from an individual carrying a homozygous *ACP4* c.746C > T, p.(P249L) variant estimated the enamel layer of a molar to be approximately 10% of the thickness of a healthy enamel layer of a matched tooth, although this was calculated from teeth that had experienced post-eruptive wear [19]. Further phenotypic studies of unerupted teeth are required to reveal the phenotypic effects of *ACP4* variants in humans.

In summary we have identified a new founder variant in *ACP4* in the South Asian population and the genetic cause of AI for 7 families. Our screening methods include use of the UK National Health Service screen for AI and a cost-effective, custom made smMIPs capture reagent, as well as exome sequencing [17]. These data suggest that biallelic *ACP4* variants account for a significant proportion of recessive AI families. We have modelled the effects of variants on the stability and structure of ACP4, showing that some variants are likely to alter protein stability while others act through different mechanisms, all of which are likely to lead to functional insufficiency. These findings provide benefits in aiding the genetic diagnosis and management of AI in the future.

## Supplementary Information

Below is the link to the electronic supplementary material.

Supplementary file1 (DOCX 205 KB)

## Data Availability

All non-identifiable sequencing data is available. Exome, Sanger, smMIPs and NHS R340 sequencing data is not available as it is deemed to be identifying. Nanopore sequencing data was uploaded to the Sequence Read Archive, available at: https://www.ncbi.nlm.nih.gov/bioproject/1403257 The data associated with this paper are openly available from the University of Leeds Data Repository: 10.5518/1814.
