# Supplementary material for: ACP4 Variants in Hypoplastic Amelogenesis Imperfecta
Source: Calcif Tissue Int. 2026 Apr 11;117(1):56. doi: 10.1007/s00223-026-01512-y (PMC13070050; doi:10.1007/s00223-026-01512-y)
Supplement: Supplementary file 1 — Supplementary file1 (DOCX 205 KB) [file 223_2026_1512_MOESM1_ESM.docx]

***ACP4* Variants In Hypoplastic Amelogenesis Imperfecta.**

Lu Liu, Cheuk Wang Au, Ummey Hany, Alice L. Rigby, Anesha Chauhan, Catriona Brown, Jessie Sims, Gina Murillo, Marìa Gabriela Acosta de Carmargo, Chris F. Inglehearn, Christopher M. Watson, Alan J. Mighell, Claire E.L. Smith.

**Supplement Figures and Tables**


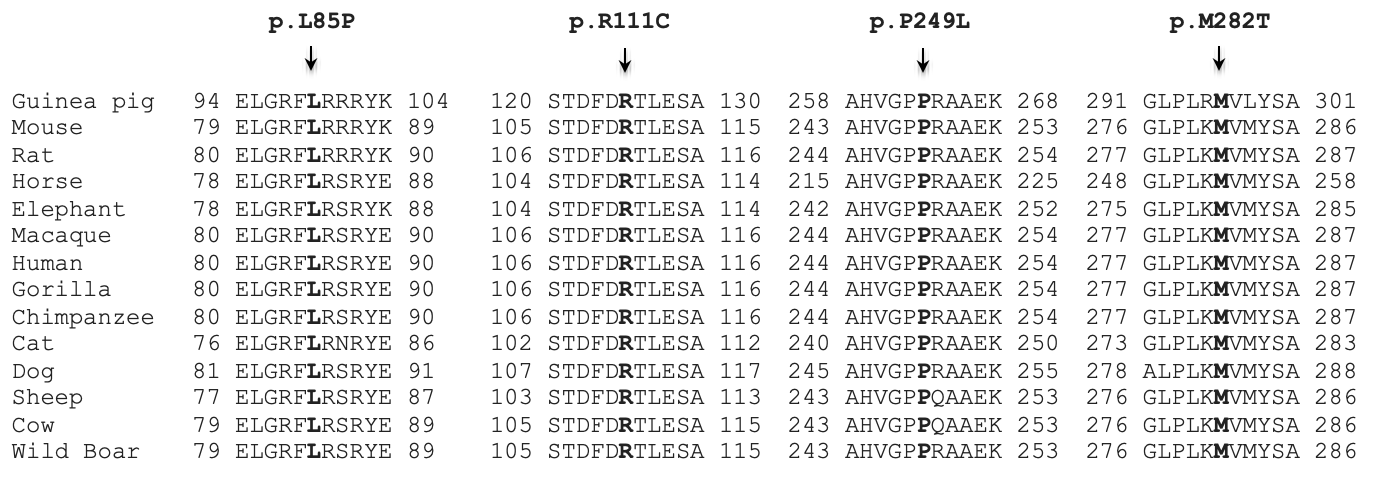
**Fig. S1** Clustal Omega multiple sequence alignment of homologues protein sequences for ACP4. The arrows indicate the altered amino acid residues substitutions across 14 species (NM_033068.3; NP_149059.1). ACPT sequences used: Guinea pig, *Cavia porcellus* XP_003467585.2; Mouse, *Mus musculus* NP_059496.1; Rat *Rattus norvegicus* NP_001099471.1; Horse *Equus caballus* XP_001487944.1; Elephant, *Loxodonta africana* XP_003414215.1; Rhesus macaque, *Macaca mulatta* XP_014994062.1; Human, *Homo sapiens* NP_149059.1; Gorilla, *Gorilla gorilla* XP_004038829.1; Chimpanzee, *Pan troglodytes* XP_526591.1; Cat, *Felis Catus* XP_003985351.1; Dog *Canis lupus familiaris* XP_539305.3; Sheep *Ovis aries* XP_004009936.1; Cow, *Bos taurus* XP_605463.4; Wild boar, *Sus scrofa* NP_999406.1.

**Table S1** Known and candidate AI genes.

|  | **Gene List** |
| --- | --- |
| Known non-syndromic AI genes | *ACP4*, *AMBN, AMELX, AMTN, COL17A1, DLX3, ENAM, FAM20A, FAM83H, GPR68, ITGB6, KLK4, LAMA3, LAMB3, LTBP3, MMP20, ODAPH, RELT, SLC24A4, SP6*, and *WDR72* |
| Known syndromic AI genes | *CLDN16, CLDN19, CNNM4, FAM20C, ORAI1, PEX1, PEX6, PEX26, PLXNB2, ROGDI, SATB1, SLC10A7, SLC13A5, STIM1* |
| NHS R340 gene panel | *ACP4, AMBN, AMELX, C4orf26, CNNM4, COL17A1, DLX3, ENAM, FAM20A, FAM20C, FAM83H, GPR68, ITGB6, KLK4, LAMA3, LAMB3, LTBP3, MMP20, ORAI1, PEX1, PEX26, PEX6, RELT, ROGDI, SLC10A7, SLC13A5, SLC24A4, SP6, STIM1, WDR72* |

**Table S2** Primers pairs for Sanger sequencing targeting identified variants.

| **Gene** | **Chromosome Location**  **(GRCh 38)** | **Primer Pairs** | **Amplicon size** |
| --- | --- | --- | --- |
| *ACP4* | chr19-50790811 | F: 5’-ATGGACCCACACAAGGAGG-3’  R: 5’-AGCGTTGTAGTCAGTGGAGG-3’ | 293 bp |
| *ACP4* | chr19-50790811 | F: 5’-ATGGACCCACACAAGGAGG-3’  R: 5’-GGTAGAGGGCAAGTCAGGTT-3’ | 350 bp |
| *ACP4* | chr19:50793784 | F: 5’-GTCCATCTGTCCTGTCTCCC -3’  R: 5’-CCGGGAGAAGTTTGCAAGG-3’ | 267 bp |
| *ACP4* | chr19-50791683 | F: 5’-CTTCCAACTTCGAAGGCCAC-3'  R: 5’- TGACCTTATCCTCAGCCACG-3' | 290 bp |
| *ACP4* | chr19-50791683  chr19-50791784 | F: 5’-TTCTGATTTGCCACGACAGC-3'  R: 5’-TGTCGCAGTTTCTCCCAGAT-3' | 397 bp |
| *ACP4* | chr19-50793954 | F: 5’-TCCTCTGGGAGAGTCTAAGCT-3'  R: 5’-ACCTAAATCGCTGTCACCCT-3' | 189 bp |

**Table S3** Benign *ACP4* variants analysed with Rosetta.

| **Genomic Position** | **Transcript Change** | **Amino Acid Change** | **CADD** | **gnomAD**  **Frequency** | **Molecular Consequence** | **dbSNP**  **Identifier** | **ACMG** |
| --- | --- | --- | --- | --- | --- | --- | --- |
| chr19-50790841 | c.284C>T | p.(Pro95Leu) | 23.2 | 0.03426 | missense | rs145475625 | Benign |
| chr19-50794569 | c.974C>A | p.(Ala325Asp) | 5.17 | 0.2905 | missense | rs55735528 | Benign |
| chr19-50794578 | c.983G>C | p.(Gly328Ala) | 7.98 | 0.01212 | missense | rs143398878 | Benign |

**Supplement Methods**

S1. PCR reaction: PCR amplification of the targeted DNA region was performed using Q5® High-Fidelity 2X Master Mix. The primer concentration was 0.5 μM and 20 ng of DNA was used for the PCR reaction. The initial denaturation step ran at 98 ˚C for 30 seconds, followed by 30 cycles of amplification (denaturation at 98 ˚C for 10 seconds, annealing at 58 ˚C for 30 seconds, and elongation at 72 ˚C for 30 seconds). The final extension was at 72 ˚C for 2 minutes then held at 4 ˚C.

S2. Nucleic acid electrophoresis: 1% agarose gel was made in TAE buffer with 4 μL Midori green added per 100 mL of TAE. 5 μL of PCR products was loaded on the gel with loading buffer, and gel electrophoresis was run at 100 V for 45 min.

S3. Sample preparation for Sanger Sequencing: 1 μL of ExoSAP-IT was used per 2.5 μL of PCR product for cleaning unused reagents in the solution. The reaction was activated at 37 ˚C for 15 min and terminated at 80 ˚C for 15 min. For 10 μL reaction mix, 1 μL of ExoSAP treated DNA, 0.75 μL of BigDye Terminator v.3.1, and 1 μL of diluted primer (1.6 μM) were prepared for sequencing. Chain termination PCR required denaturation at 96 ˚C for 1 minute, followed by 25 cycles of amplification (96 ˚C for 10 seconds, 50 ˚C for 5 seconds, and 60 ˚C for 4 minutes) with all temperatures ramped at 1 ˚C/sec. Samples were held at 12 ˚C.

S4. DNA precipitation: 10 μL of the chain termination reaction product was transferred to a 96-well plate for Sanger sequencing. 5 μL of 125 mM EDTA and 60 μL of 100 % ethanol were added in sequence and the solution was kept at -20 ˚C for at least 15 min. The mixture was centrifuged at 3900 rpm for 30 min at 20 ˚C and the liquid solution was removed. 60 μL of freshly prepared 70% ethanol was added to each sample and centrifuged at 2000 rpm for 15 min at 4 ˚C. The liquid solution was removed and the plate was dried at 37 ˚C for 2 min to remove residual ethanol. 10 μL of HiDi Formamide was added to each well to dissolve the pellet for Sanger sequencing.
